# *Methylobacterium* spp. Isolated From Semiarid Soils Promote Growth and Drought Tolerance in Maize in Kenya

**DOI:** 10.1155/ijm/7442350

**Published:** 2025-09-08

**Authors:** Emmanuel Ehinmitan, Beenzu Siamalube, Turoop Losenge, Edward Mamati, Patrick Juma, Victoria Ngumi

**Affiliations:** ^1^Department of Molecular Biology and Biotechnology, Pan African University Institute for Basic Sciences, Technology and Innovation, Nairobi, Kenya; ^2^Department of Horticulture, Jomo Kenyatta University of Agriculture and Technology, Nairobi, Kenya; ^3^Department of Botany, Jomo Kenyatta University of Agriculture and Technology, Nairobi, Kenya

**Keywords:** antioxidant activity, drought tolerance, maize, *Methylobacterium* spp., PGPR, rhizosphere

## Abstract

Drought significantly impacts crop growth and yield. Advances in agricultural biotechnology have enabled the use of drought-tolerant bacterial strains to mitigate the detrimental effects of drought on crops. This study, conducted during the 2024 dry season in Kiambu County, Kenya, evaluated 100 methylotrophic bacteria isolated from the rhizosphere of maize variety WE-4141 cultivated under severe drought conditions. Isolation was performed via serial dilution using ammonium mineral salt (AMS) medium supplemented with methanol as the sole carbon source, with identification confirmed through 16S rRNA gene sequencing. The isolates were assessed for plant growth–promoting and drought-tolerance properties under varying polyethylene glycol (PEG) 6000–induced osmotic stresses (−0.13, −0.50, −0.75, and −1.3 MPa). Seed priming was used to introduce bacterial inoculants at 10^8^ CFU/mL concentration. Under PEG-induced stress, strains K19 and K2 produced the highest quantities of gibberellic acid (GA) and indole acetic acid (IAA), with K2 also showing superior exopolysaccharide (EPS) production at the highest osmotic level. Enhanced biofilm formation was observed under drought conditions compared to controls. Antioxidant enzyme activity correlated positively with increasing PEG concentrations, with K19 exhibiting the highest catalase activity. In planta, drought stress significantly reduced plant growth, with uninoculated controls showing notable decreases in plant height, root, and shoot dry weights. Inoculation with K2 and K19 enhanced maize performance under drought, with their combination yielding the greatest improvements: shoot dry weight increased by 42.0%, root dry weight by 46.2%, and plant height by 65.8% compared to controls. These findings suggest that strains K2 and K19 hold potential as biostimulants for improving maize resilience and productivity under drought conditions and warrant further evaluation through field trials for potential bioformulation development.

## 1. Introduction

Globally, drought conditions have become increasingly frequent and unpredictable. This stress has negatively impacted the growth and yields of economically important crops. The increasing severity of droughts driven by climate change has further worsened the challenges faced by traditional agricultural practices [1]. In Kenya, maize (*Zea mays* L.) remains a vital staple crop, contributing approximately 57% of daily caloric intake and supporting the livelihoods of over 6 million people. In 2021, Kenya harvested 4.38 million tons of maize from over 2.55 million hectares, yielding an average of 22.8 bags (90 kg) per hectare, valued at Ksh 199 billion. Despite this, Kenya remains a maize-deficit country, with recent imports exceeding 488,000 tons to supplement domestic consumption [2]. Key maize-growing counties include Uasin Gishu, Trans Nzoia, Bungoma, Narok, and Machakos [3]. Maize productivity in Kenya is particularly vulnerable to drought stress, which remains a significant constraint on agricultural output and food security. Drought events, such as the 2008–2010 drought that affected approximately 10 million people, continue to reduce yields, particularly in Kenya's arid and semiarid regions where maize losses can reach 30%–60% in drought years [4].

Maize is a staple grain of immense dietary significance, playing a crucial role in addressing hunger for billions worldwide, and its productivity is heavily impacted by drought stress [5]. The crop is particularly vulnerable to drought during its tasseling and silking developmental stages, which hamper growth and reduce grain yield [6]. Drought causes water scarcity in the crop's root microenvironment, soil degradation, and biodiversity loss, affecting food security and livelihoods. The reliance on genetic modification and classical breeding has provided some relief but has not fully addressed the multifaceted nature of drought stress on crops.

In response to these limitations, the focus has shifted to more holistic and environmentally sustainable approaches to enhance productivity and plant tolerance in drought stress conditions. One promising solution lies in exploiting plant growth–promoting bacteria (PGPB), which are often found in rhizospheres, rhizoplanes, phyllospheres, or endospheres. These beneficial microbes offer a sustainable alternative to chemical fertilizers. PGPB directly improves plant growth through enzymatic activities [7], the production of 1-aminocyclopropane-1-carboxylic acid (ACC) deaminase that adjusts levels of ethylene in plants [8] and enhancing the availability of phosphorus and the production of siderophores that chelate iron [9].

PGPB's role extends beyond direct plant growth promotion. They also indirectly improve plant development by reducing the detrimental impacts of plant-targeting pathogens, including bacteria, fungi, nematodes, and viruses [10]. This dual action makes PGPB an attractive option for integrated pest management (IPM) strategies, which aim to reduce reliance on chemical pesticides and mitigate their negative environmental impacts. Furthermore, studies have highlighted the role of PGPB in providing an economical and ecofriendly approach to counteract drought stress in various plants [11].

The application of PGPB as bioinoculants in agriculture is not without its challenges. The relationship between plants and PGPB is intricate and varies with the genetic compatibility of PGPB strains and the crops. Hence, to improve agricultural yields through microbial interactions, it is important to select the most suitable PGPB strains for specific agricultural conditions [12]. The specificity of these interactions means that a strain beneficial for one crop might not be as effective for another. This necessitates extensive field trials and research to identify and optimize PGPB applications tailored to different crops and environmental conditions.

Among the various PGPB, pink-pigmented facultative methylotroph (PPFM) bacteria within the *Methylobacterium* genus are known for their high production of active plant hormones. These bacteria can synthesize cytokinins, auxins, and gibberellins, which are critical for plant growth and stress tolerance [13]. The PPFM bacteria's ability to utilize methanol, a byproduct of plant metabolism, further enhances their symbiotic relationship with plants, making them an efficient and self-sustaining option for improving crop resilience under drought conditions [14].

PPFM are capable of metabolizing other single-carbon energy sources such as methylamine [15]. Their interactions with plants range from free-living to symbiotic relationships. *Methylobacterium* spp. contribute to the improvement of seed germination, enhance seedling growth, and promote systemic defense across a wide range of plants [16]. Foliar application of PPFMs on strawberries elevated growth and development [17], while in rice, an increase in root development was observed [18]. Furthermore, they enhance seed germination and growth in maize [19] and potato [20]. In legumes, *Methylobacterium* spp. increased both pod and seed mass [21]. PPFMs are known to alleviate drought stress in tomatoes [22].

Despite these promising findings, location-specific strains of *Methylobacterium* with potential as bioinoculants for maize cultivation in arid and water-limited environments remain unidentified and untested. While much research has focused on established genera like *Bacillus* and *Rhizobium*, there is a growing need to diversify the exploration of beneficial microbes suited to the unique agroecological conditions of Kenya. Therefore, this study is aimed at isolating *Methylobacterium* strains from semiarid areas in Kenya and evaluating their capability to enhance maize growth under drought stress conditions.

## 2. Materials and Methods

### 2.1. Sampling Site

Maize rhizosphere soil samples were collected (March 2023) from Makueni County in Kenya (−2 01°60.00⁣′ S and 37.42°21⁣′59.99⁣′ E). The plant was chosen based on its known adaptation to semiarid climatic conditions, with an annual rainfall range of 250–400 mm. Slight agitation was employed to remove loose soil, followed by brushing to collect the remaining rhizosphere soil. Sterile techniques were used to avoid contamination. The sampling area has been used to grow maize continuously.

### 2.2. Isolation of *Methylobacterium* spp.

Serially diluted soil samples were plated on ammonium mineral salt (AMS) media agar supplemented with methanol as the sole carbon source (March 2023, −1° S 1°6.2371⁣′ E 37°0.8997⁣′). Cycloheximide (50 *μ*g/mL) and nalidixic acid (100 *μ*g/mL) were added to prevent the growth of fungi. The plates were incubated at 28°C for 5 days, and distinct colonies were purified using the same isolation medium. Pure isolates were stored in 60% glycerol at −60°C for future use. Subsequently, the isolates were subjected to drought-induced stress. This was done by introducing polyethylene glycol (PEG) 6000 into AMS + methanol broth, followed by inoculation with a loopful of the pure colony. PEG 6000 osmotic potentials (Ψ) were calculated using empirical relationships outlined by Michel and Kaufmann [23]. After a 120-h incubation period at a temperature of 28°C, the growth of the isolates was evaluated by measuring the optical density (OD_600_) using a spectrophotometer (Jenway, M6000, United Kingdom) with sterile methanol supplemented AMS + PEG 6000 used as the blank. The isolates were classified according to their optical density (OD_600_) values, which were used to assess their degree of drought sensitivity. Isolates with an OD_600_ value less than 0.3 were categorized as highly sensitive, those with an OD_600_ value between 0.3 and 0.39 were classified as sensitive, isolates with an OD_600_ value between 0.4 and 0.5 were considered tolerant, and isolates with an OD_600_ value greater than 0.5 were classified as completely tolerant [24].

### 2.3. Characterization of Plant Growth–Promoting (PGP) Properties of the *Methylobacterium* spp.

#### 2.3.1. Preparation of Cell-Free Supernatant

Tubes containing AMS medium were set up under nonstressed and stressed conditions at varying water potentials (−0.13, −0.5, −0.75, and −1.3 MPa). A loopful of inoculum from the fresh cultures of highly drought-tolerant isolates was added, and the tubes were incubated at 28°C for 120 h in a shaking incubator at 150 rpm. Afterward, the cultures were centrifuged at 10,000 rpm for 5 min to separate the supernatant, which was collected as a cell-free supernatant for subsequent assays [24].

#### 2.3.2. Indole Acetic Acid (IAA) Biosynthesis

IAA production was quantified by adopting the protocol described by Bric et al. [25]. An inoculum constituting 10% of bacterial culture in the exponential growth phase was introduced into 100 mL methanol-supplemented AMS broth. L-Tryptophan at a concentration of 100 *μ*gmL^−1^ was incorporated as the precursor for IAA synthesis. After 120 h of incubation, the bacterial cell-free supernatant was harvested for use in the quantitative analysis of IAA. For the assay, 1 mL of the obtained supernatant was vigorously mixed with 1 mL of Salkowski's reagent (prepared by dissolving 1 mL of 0.5 M ferric chloride [FeCl_3_] into 50 mL of 35% HClO_4_). Subsequently, two drops of H_3_PO_4_ were added. Next, the mixture was incubated in the dark at a temperature of 28°C for 20 min. The pink hue formation depicts a positive test, and the intensity of this coloration was assessed at a wavelength of 535 nm. A preestablished standard curve for IAA was used to determine the concentration of IAA in each sample.

#### 2.3.3. Production of Exopolysaccharide (EPS)

The bacterial isolates were cultured using 50 mL of mineral salt medium. The medium consisted (*w*/*v*) of 12.6% dipotassium hydrogen phosphate, 18.2% potassium dihydrogen phosphate, 10% ammonium nitrate, 1% magnesium sulfate heptahydrate, 0.6% manganese (II) sulfate, 1% calcium chloride dihydrate, 0.06% iron (II) sulfate heptahydrate, 1% sodium molybdate dihydrate, 1.5% sodium chloride, and 0.5% methanol. The approach used in this study was a modification of the methods described by Naseem and Bano [26]. The incubation process was carried out at a temperature of 28°C for 10 days under both stress and no-stress conditions in a shaker incubator at a speed of 150 rpm. Next, the cells were collected by adding 0.5 mL of 1 mM EDTA, and the mixture was vigorously agitated. Subsequently, the mixture was centrifuged at 15,000 rpm for 10 min. The supernatant was separated and combined with a 95% ethanol biphasic solution, then cooled to freezing temperatures. The centrifugation process was done twice, each iteration lasting 30 min at the same speed. The precipitates were then collected, thoroughly washed, and dried. The last stage was the measurement of the weight of the sample in its dried state.

#### 2.3.4. Production of Gibberellic Acid (GA)

In a test tube, 1000 *μ*L of bacterial-free supernatant was mixed with 1000 *μ*L of Folin–Ciocalteu reagent and 1000 *μ*L of 12 M hydrochloric acid (HCl). Afterward, 3000 *μ*L of distilled water was added. Next, the mixture was subjected to hydrothermal treatment for 300 s. Subsequently, it was left to cool. The positive test bluish-green hue was quantified using a spectrophotometer at a wavelength of 760 nm. The quantification of GA generated by the isolates was done using the GA standard solution [27].

### 2.4. Characterization of Drought Tolerance Properties of the *Methylobacterium* spp.

#### 2.4.1. Proline Production

A reaction mixture was prepared by combining 1.5 mL of the bacterial supernatant with equal glacial acetic acid. Then, 1.5 mL of acid ninhydrin reagent was added. The ninhydrin reagent was prepared by dissolving 2.5 g of ninhydrin in a solvent mixture containing 60 mL of glacial acetic acid and 40 mL of 6 M phosphoric acid, which was then heated until dissolution. Next, the resulting mixture was put into a glass tube and agitated using 3.0 mL of toluene for 20 s to achieve homogeneous mixing. Blank mixes without bacterial inoculum were used for absorbance measurements at 520 nm. Proline quantification was done using a standard curve, with purified proline concentrations used to plot and generate the equation of the line required in concentration quantification [24].

#### 2.4.2. Salicylic Acid (SA) Production

The measurement of SA produced by the isolates was done following the protocol established by El-Meihy et al. [27]. The procedure involved adjusting the pH of a 4.0 mL aliquot of bacterial cell-free supernatant to 2. To achieve this, 1 N HCl was gradually added to the aliquot to decrease the pH. The acid extraction was done using chloroform (CHCl_3_) in a 1:1 volume ratio. Next, 4.0 mL of distilled water, followed by 5.0 mL of a 2 M solution of FeCl_3_, was added to the mixture. The spectrophotometric analysis measured the absorbance at 527 nm using a mixture without bacterial inoculum as a blank. SA quantification was done using a standard curve, with purified SA concentrations used to plot and generate the equation of the line required in concentration quantification.

#### 2.4.3. Antioxidant Enzyme Activity

##### 2.4.3.1. Catalase (CAT)

The CAT activity was quantified by measuring the decrease in optical density at 240 nm, which is a reliable indicator of hydrogen peroxide (H_2_O_2_) breakdown. This procedure followed the methodology described by Desoky et al. [28]. The setup consisted of 500 *μ*L of a 75 mM H_2_O_2_ solution, 1500 *μ*L of a potassium phosphate buffer with a concentration of 100 mM and a pH of 7.0, 200 *μ*L of the enzymatic extract, and 800 *μ*L of distilled water. CAT quantification was done using a standard curve, with purified CAT concentrations used to plot and generate the equation of the line required for concentration quantification.

##### 2.4.3.2. Peroxidase (PO)

To evaluate PO enzymatic activity, 4-methyl catechol was utilized using the protocol described by Onsa et al. [29]. A mixture was prepared with distilled water containing 100 mL of potassium phosphate buffer (100 mM, pH 7.0), 500 mL of H_2_O_2_ (5 mM), 500 mL of 4-methyl catechol (5 mM), and 500 mL of the crude enzyme extract, with the total volume adjusted to 4000 mL. Absorbance measurements were conducted at 420 nm. For this, enzyme activity was quantified as the amount of enzyme necessary to produce a 0.001 absorbance unit change per minute. PO quantification was done using a standard curve, with purified PO concentrations used to plot and generate the equation of the line required in concentration quantification.

##### 2.4.3.3. Polyphenol Oxidase (PPO)

PPO activity was assessed using the methodologies outlined by Oktay et al. [30]. The experimental procedure included the mixture of 100 *μ*L of a 100 mM sodium phosphate buffer with a pH of 7.0, 500 *μ*L of a 5 mM 4-methyl catechol solution, and 500 *μ*L of crude enzyme extract. Subsequently, 2900 *μ*L of distilled water was introduced into the resulting mixture. Spectrophotometry was used to assess the absorbance at a wavelength of 420 nm. In this experimental setting, a single enzyme activity unit is defined as the enzyme's ability to cause a change in absorbance of 0.001/min. PPO quantification was done using a standard curve, with purified PPO concentrations used to plot and generate the equation of the line required in concentration quantification.

### 2.5. Molecular Identification and Phylogenetic Assessment of the *Methylobacterium* spp.

#### 2.5.1. Extraction of Bacterial Genomic DNA and Polymerase Chain Reaction (PCR) Amplification

The bacterial genomic DNA was extracted using the ZymoBIOMICS DNA Miniprep kit, following the manufacturer's recommended methodology. Following DNA isolation, the Universal 16S rRNA gene was amplified using specific primers, notably 27F (5⁣′-AGAGTTTGGATCCTGGCTCAG-3⁣′) and 1492R (5⁣′-CGGTTACCTTGTTACGACTT-3⁣′). The PCR was performed using a thermal cycler (ProFlex, Singapore) and a reaction mixture volume of 50 *μ*L. The composition consisted of a 0.4 *μ*M concentration of each primer, a 400 *μ*M concentration of dNTP mix, 5 *μ*L of PCR-specific buffer, 2 *μ*M MgCl_2_, 2.5 units of Taq DNA polymerase (Sigma-Aldrich, D1806), 1 *μ*L of the genomic template, and sterile nuclease-free water to adjust the volume. The PCR started with an initial denaturation phase at 95°C for 3 min. This was followed by 35 cycles of denaturation at 95°C for 50 s, annealing at 55°C for 60 s, extension at 72°C for 1 min, and a final extension step at 72°C for 10 min [31].

#### 2.5.2. Sequencing and Phylogenetic Analysis of DNA

DNA amplicons following PCR amplification were subjected to a purification process utilizing a QIAquick purification kit (United States), adhering strictly to the protocol provided by the kit's manufacturer. Subsequently, sequencing was performed. The BLAST algorithm was done to compare the generated 16S rRNA sequences with those deposited in the NCBI nucleotide database. This comparison aims to determine closely related bacterial species. The sequence alignment was done with CLUSTALW, and the neighbor-joining method was utilized to determine the phylogenetic relationships using MEGA X software Version 11.0.13 [32].

### 2.6. Evaluation of Biofilm Biosynthesis Under Drought Conditions

The biofilm-forming capacity of the isolates was assessed under PEG 6000 stress (−1.3 MPa) and no-stress (0 MPa) control conditions. This was accomplished using the colorimetric approach, as outlined by Ashry et al. [24]. The selection of −1.3 MPa ensures a balance between inducing stress responses and maintaining cellular viability [33]. Log phase cultures were loaded into individual microtiter plate wells at a final concentration of 10^4^ CFU/mL. The cultures were inoculated with methanol-supplemented AMS without PEG 6000 to simulate nonstressed conditions. The Petri dishes underwent incubation at 28°C for 120 h. The evaluation of biofilm formation was carried out using quantitative analysis, which involves the measurement of absorbance at 570 nm [34].

### 2.7. Assessment of Antioxidant Properties and Microbial Biosurfactant Production

#### 2.7.1. Evaluation of Antioxidant Properties

Five hundred microliters of cell-free supernatant was combined with 3000 *μ*L of a freshly prepared solution containing 2,2-diphenyl-1-picrylhydrazyl (DPPH) at a concentration of 0.1 mM. This mixture was then allowed to incubate for 30 min in darkness. A control sample was prepared by adding 500 *μ*L of ethanol to 3000 *μ*L of the DPPH solution. Following the incubation period, the absorbance was determined at a wavelength of 517 nm using a spectrophotometer (Jenway, M6000, United Kingdom). The percentage of radical scavenging activity was subsequently calculated using the following formula [35]:

(1) $$\begin{array}{c} \%\text{of DPPH Radical Scavenging Activity}=\frac{\left(\text{OD of the control}-\text{OD of the treated sample}\right)}{\left(\text{OD of the control}\right)}\times 100. \end{array}$$

#### 2.7.2. Production of Biosurfactant

The emulsification capacity was quantified, denoted as emulsification index after 24 h (%EI 24). This was achieved by mixing equal volumes of the culture's supernatant with toluene in a clear glass tube, subjecting it to 2 min of vigorous vortex agitation. Next was a rest period of 24 h without disturbance. The %EI 24 was calculated using the following formula [36]:

(2) $$\begin{array}{c} \%\text{EI }24=\frac{\text{Height of the resulting emulsion }}{\text{Total solution height}}\times 100. \end{array}$$

### 2.8. Assessment of Seed Biotization Efficiency

The biotization efficiency of *Methylobacterium* spp. on drought-susceptible maize seed was determined both with and without −1.3 MPa PEG 6000-induced stress conditions [37]. Maize seeds susceptible to drought were sourced (DeKalb, Kenya) and surface sterilized by immersing them in a solution of 70% ethanol for 5 min, followed by a 15-min exposure to a 2% sodium hypochlorite (NaClO_2_) solution. After these treatments, the seeds were thoroughly washed with sterile distilled water to eliminate residual sterilizing agents. For the experimental setup, two filter papers were placed inside each of several Petri dishes, and each treatment was in triplicate. To each dish, 10 mL of bacterial suspension grown in −1.3 MPa of PEG 6000 was added. In contrast, 10 mL of distilled water was used for control samples. Fifteen sterilized seeds were placed in each dish and incubated at 25°C for 10 days. Prior to this, a 5-h seed presoaking in a 10 mL solution containing bacterial strains was done, with and without the addition of PEG, in a rotary shaker set at 150 rpm. The number of seeds that germinated in each dish was recorded. Three seedlings were randomly selected from each dish to measure the overall seedling length. The germination percentage and the vigor index were calculated using a method outlined by Chukwuneme et al. [38], detailed as follows:

(3) $$\begin{array}{c} \text{Germination efficiency}\%=\frac{n\;}{N}\times 100, \end{array}$$

where *n* is the count of seeds that germinated by Day 7 and *N* represents the initial seed count.

(4) $$\begin{array}{c} \text{vigor index}=\text{germination percentage}\times \text{aggregate length of the seedlings}. \end{array}$$

### 2.9. In-Planta Effects of Drought Conditions and *Methylobacterium* spp. Inoculation on Maize Growth

To assess the effects of inoculating maize plants with *Methylobacterium* spp. on mitigating drought stress, five distinct treatments were developed. These treatments included both individual and combined inoculations of the two most effective strains (K2 and K19), alongside control groups under both watered and drought conditions. The control groups consisted of a “watered” uninoculated control, maintained at full field capacity without drought stress, and a “drought” uninoculated control, which was subjected to 33 days of drought at 30% field capacity. Additionally, the other three treatments involved inoculating maize seeds with K2, K19, and a combination of the two strains, with the same 33-day drought period. The experiments were carried out in a greenhouse environment using a completely randomized block design, with five replicates for each treatment, all conducted under semicontrolled conditions.

The maize seeds underwent surface sterilization by immersing them in 5% (*v*/*v*) NaClO_2_ for 3 min, followed by treatment with 70% (*v*/*v*) ethanol for 2 min, and were then rinsed three times with sterile water. A single colony of each bacterial strain was cultured in AMS medium, incubated for 120 h at 28°C, and subjected to shaking at 150 rpm. After incubation, the colony-forming units per milliliter were quantified, and the cell suspension was adjusted to a concentration of 1 × 10^8^ CFU mL^−1^ using saline solution. Seed inoculation involved immersing the sterilized seeds for 2 h in the previously standardized cell suspension of each strain while shaking at 200 rpm. The pots were filled with 1.5 kg of autoclaved cocopeat, which was adjusted to 70% of its field capacity. Three seeds were sown per pot, and after 12 days of germination, only one plant was retained per pot. The plants were maintained at 30% of field capacity until they reached 45 days of growth. The plant growth medium was supplemented with Hoagland Basal Salt Mixture in distilled water, and measurements of plant height, shoot, and root dry weight were subsequently taken [39].

### 2.10. Statistical Analysis

The experimental procedures were replicated three times. Data on proportions (percentage) were arcsine square root transformed before being subjected to any statistical analysis. Treatment effects were determined using analysis of variance (ANOVA) only when variance homogeneity could be assumed. When variance was heterogeneous, or in the case of unequal sample sizes, data were analyzed using the generalized linear models (GLMs). Whenever the treatment effects were significant, means were compared using Tukey's honest significant difference test with the SPSS 14.0 software. All tests were performed at the 5% level of significance.

## 3. Results

**Figure 1:**
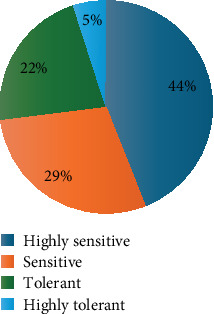
Percentages of tolerance levels of bacterial isolates on methanol + AMS medium fortified with PEG 6000 (−1.3 MPa).

One hundred methylotrophic bacteria were isolated from the maize rhizosphere grown in the semiarid climatic conditions of Makueni County of Kenya. When the isolates were subjected to conditions of high drought stress (−1.3 MPa), 44% of them could not withstand the conditions and were classified as highly sensitive, 29% were sensitive, 22% were tolerant, and 5% were highly tolerant (Table S1). The five highly tolerant isolates (MK31, K2, K19, SH26, and SH33) were selected as elite drought-tolerant isolates for subsequent assays (Figure 1). The sensitivity of the bacterial isolates to osmotic pressure is significantly varied (*p* < 0.05), depicting notable differences in tolerance levels among the isolates.

### 3.1. Plant Growth–Stimulating Properties of *Methylobacterium* spp.

**Figure 2:**
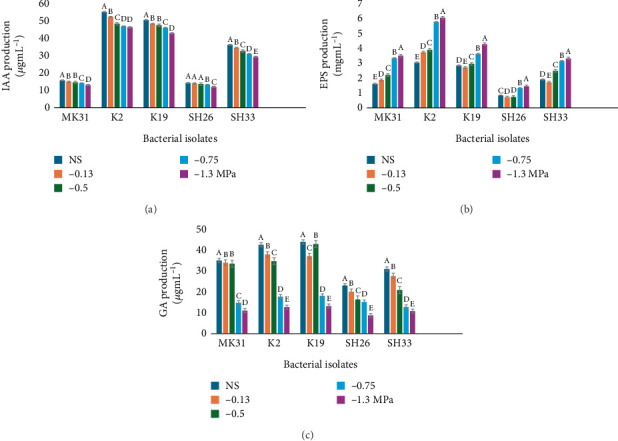
The isolates production of (a) IAA, (b) EPS, and (c) GA at different stress levels (−0.13, −0.5, −0.75, and −1.3 MPa) and no-stress conditions (NS) as control. The presented data represent average measurements obtained from triplicate experiments, each with its standard deviation (±SD). When means share identical letters, it indicates no significant differences at a significance level of 0.05, as assessed by the Tukey test.

The five elite isolates in terms of in vitro drought stress tolerance were evaluated for their potential ability to enhance plant growth. Bacterial PGP properties tested were IAA, GA, and EPS production under varying conditions of drought stress. The trends in producing these compounds in response to varying drought stress treatments were observed and presented in Figure 2. The production levels of IAA biosynthesis in all the isolates were significantly high under no-stress conditions. However, the quantity of IAA produced under stress varied, with isolate K2 leading in IAA synthesis, with a production range (46.45–55.37 *μ*g/mL). K19 closely followed this, whereas SH26 was the lowest producer of IAA, with levels ranging from 12.04 to 14.26 *μ*g/mL (Figure 2a).

The EPS production increased with the severity of drought stress (Figure 2b). Isolate K2 was the best in terms of EPS production (6.07 mg/mL). Isolate K19 also showed notable EPS productivity under elevated stress conditions, whereas SH26 exhibited the lowest EPS output. The elevation in EPS production across all the isolates under drought stress typifies the critical role of EPS in mitigating drought stress impact. Furthermore, an inverse relationship was observed between the concentration of PEG 6000 and GA production, with K19 emerging as the most efficient GA producer, presenting a range from 13.22 to 44.16 *μ*g/mL (Figure 2c). This was closely followed by K2, while SH26 was identified as the least efficient, producing GA within a range of 8.70–23.12 *μ*g/mL.

### 3.2. Drought Tolerance Properties of the *Methylobacterium* spp.

**Figure 3:**
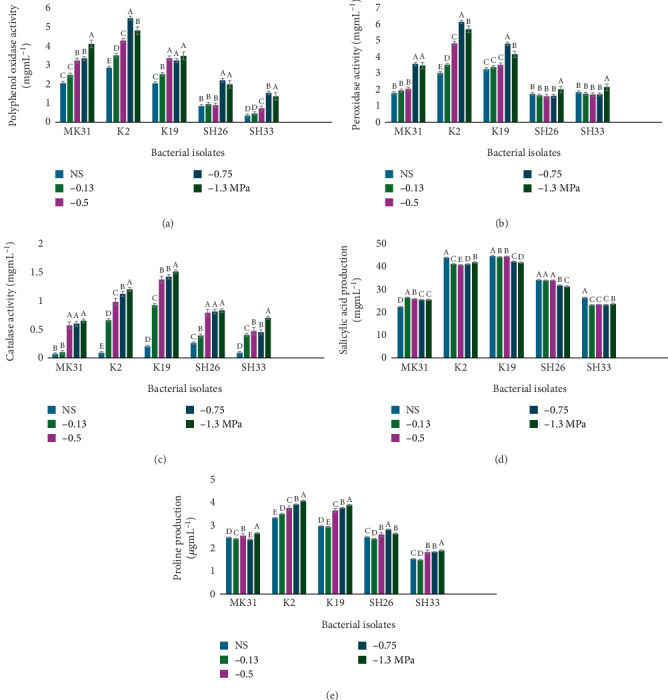
The isolates production of (a) PPO, (b) PO (c) CAT, (d) SA, and (e) proline at different stress levels (−0.13, −0.5, −0.75, and −1.3 MPa) and no-stress conditions (NS) as control. The presented data represent average measurements obtained from triplicate experiments, each with its standard deviation (±SD). When means share identical letters, it indicates no significant differences at a significance level of 0.05, as assessed by the Tukey test.

The activities of PPO and PO in all the tested isolates depended on the varying levels of PEG 6000 (Figure 3a,b). Strain K2 exhibited the highest PPO activity, with K19 showing notable PO activity and MK31 for PPO activity. Additionally, CAT activity in the isolates increased with an increase in PEG 6000 concentration, reaching its highest at −1.3 MPa stress level. Isolate K19 had the highest CAT activity (1.51 mg/mL), whereas strain MK31 exhibited the lowest activity, 0.01 mg/mL, under no-stress conditions (Figure 3c).

SA production varied among all isolates produced, with differing amounts under both stress and no-stress conditions (Figure 3d). Isolates K19 and SH33 were the highest and lowest SA producers, respectively. Notably, K2 produced significantly more SA (41.75–43.85 mg/mL) than SH26 and MK31. Proline levels in the isolates increased with higher PEG 6000 concentrations. Isolate K2 had the highest proline levels, ranging from 3.33 to 4.07 mg/mL under stress and control, respectively. The lowest proline production was in isolate SH33, with levels ranging from 1.53 to 1.90 mg/mL (Figure 3e). Based on the results obtained in the preliminary testing of the isolates for PGP and drought-tolerant properties, isolates K2 and K19 were selected as the two elite isolates for further studies.

### 3.3. Identification and Phylogenetic Characterization of Bacterial Strains

**Figure 4:**
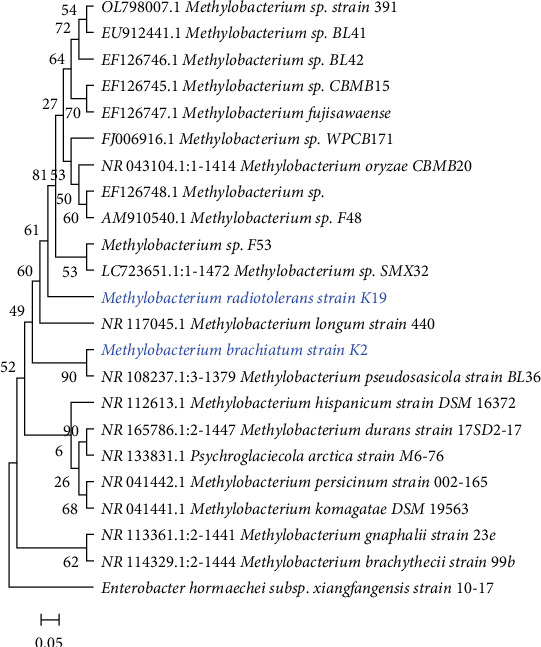
A phylogenetic analysis using the neighbor-joining method based on 16S rRNA gene sequences, aligning each sequence with its nearest phylogenetic counterparts. The reliability of the tree's branches is evaluated through bootstrap testing, with values presented at the branching points to indicate confidence levels.

Amplification and sequencing of the 16S rRNA gene fragments, each spanning about 1.5 kb, were performed using PCR for two isolates. This was to ascertain the specific identity of each isolate by analyzing segments of the 16S rRNA gene. This analysis identified the isolates as strains of *Methylobacterium brachiatum* (K2) and *Methylobacterium radiotolerans* (K19). The sequences derived from this analysis were deposited in the NCBI GenBank database, receiving the Accession Numbers PP708908 for the *M. brachiatum* K2 and PP708910 for the *M. radiotolerans* K19. The outcomes of conducting a BLAST search compared these sequences against those previously deposited in the GenBank database (Table 1). Furthermore, the phylogenetic tree, which was constructed using the neighbor-joining method, highlighted the evolutionary relationships between the sampled *Methylobacterium* strains and other representative species, drawing on comparisons of partial 16S rRNA gene sequences (Figure 4).

### 3.4. Evaluation of Biofilm Formation by the Elite *Methylobacterium* Strains Under Drought Stress Conditions

**Figure 5:**
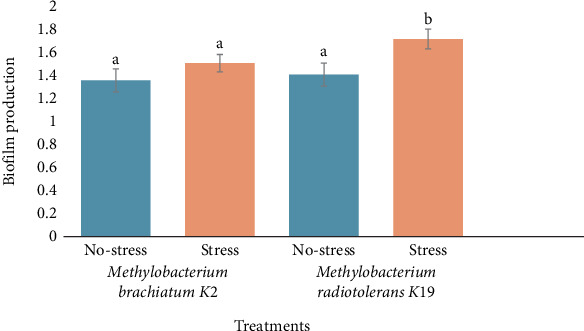
Biofilm production by K2 and K19 strains in stress and no-stress conditions. The presented data represent average measurements obtained from triplicate experiments, with error bars indicating the deviations in the values. Identical letters above the means signify no significant differences between them at a 0.05 significance level, as determined by the Tukey test.

Biofilm production varies between *M. brachiatum* K2 and *M. radiotolerans* K19 under stress and no-stress conditions. Specifically, strain K2 displayed no significant difference in biofilm production between no-stress and stress conditions. K2 maintains a consistent level of biofilm formation regardless of induced drought stress. In contrast, *M. radiotolerans* K19 exhibited a significant increase in biofilm production under stress conditions. The biofilm production level under stress for strain K19 was significantly higher than that under no-stress conditions. This result suggests that K19 responds to stress by enhancing biofilm production, potentially as a survival mechanism (Figure 5).

### 3.5. Evaluation of Antioxidant and Biosurfactant Properties

**Figure 6:**
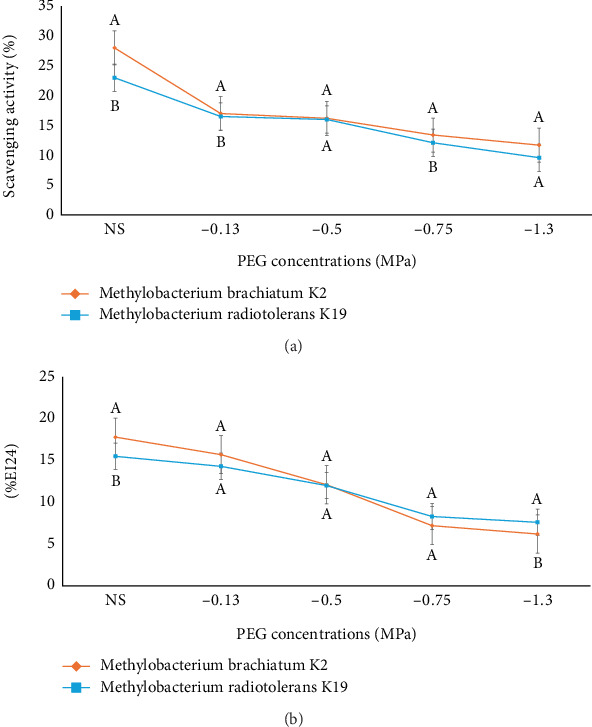
Percentage of DPPH (a) scavenging activity and (b) biosurfactant production of strains K2 and K19 under stress and no-stress conditions. The presented data represent average measurements obtained from triplicate experiments, with the error bars showing the deviation in the values.

The K2 and K19 strains were further tested for their ability to scavenge DPPH radicals and to produce biosurfactants under drought and no-stress conditions. It was observed that the ability of the strain to neutralize DPPH radicals decreased with increasing concentration of PEG 6000. Notably, K2 showed weaker DPPH scavenging efficiency in comparison to K19 (Figure 6a), indicating that K2 has superior effectiveness in DPPH inhibition. The oil emulsification method was employed to evaluate the strains' biosurfactant production (Figure 6b). Under no-stress conditions, K2 and K19 attained emulsification indexes of 17.84% and 15.56%, respectively, which decreased under stress conditions.

### 3.6. Seed Biotization Efficiency Test

The application of *M. brachiatum* K2 and *M. radiotolerans* K19 enhanced the maize seed germination rate, seedling growth, and overall vigor in no-stress conditions (T7). However, under conditions of drought stress, seeds treated with both K2 and K19 (T8) demonstrated enhanced performance in germination rate, length of seedlings, and vigor index, achieving the highest recorded values of 80% for germination, seedlings measuring 9.6 cm in length, and a vigor index of 768 in comparison to treatments (T2, T4, and T6) with either or none of the inoculants (Table 2).

The presented data represent average measurements obtained from triplicate experiments at −1.3 MPa osmotic stress pressure, each with its standard error (±SE). When means share identical letters, it indicates that there are no significant differences between them at a significance level of 0.05, as assessed by the Tukey test.

### 3.7. In-Planta Effects of Drought Conditions and *Methylobacterium* spp. Inoculation on Maize Growth

**Figure 7:**
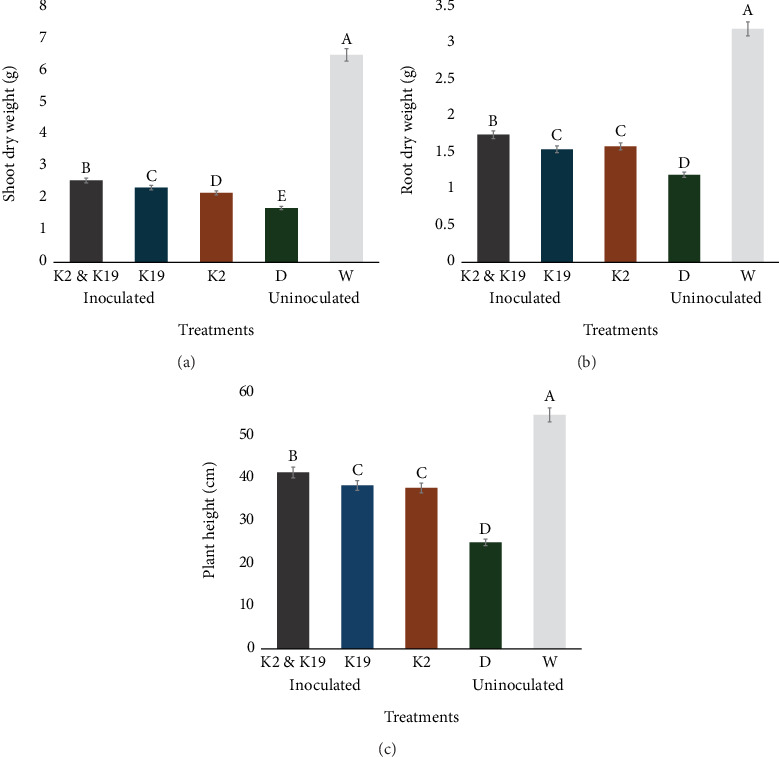
Effect of *Methylobacterium* spp. inoculation on maize crop growth under watered and drought conditions of (a) shoot dry weight, (b) root dry weight, and (c) plant height. The presented data represent average measurements obtained from triplicate experiments, with error bars indicating the deviations in the values. Identical letters above the means signify no significant differences between them at a 0.05 significance level, as determined by the Tukey test.

Drought stress led to a notable decline in plant growth, with substantial reductions in plant height (60.0%), root dry weight (72.6%), and shoot dry weight (62.0%) in plants that were not inoculated (Figures 7a, 7b, and 7c). However, inoculation with *Methylobacterium* spp. helped mitigate these effects, increasing total biomass in drought-affected plants. Specifically, strain K2 boosted shoot dry weight by 28.0%, K19 by 39.0%, and the combination of K2 and K19 by 42.0%, compared to the stressed, uninoculated controls (Figure 7a). Additionally, the combined inoculation of K2 and K19 resulted in the most significant improvement in root dry weight (46.2%), followed by K2 (32.5%) and K19 (28.9%), compared to the drought-stressed, uninoculated plants (Figure 7b). In terms of plant height, the combination of K2 and K19 led to a 65.8% increase, with K2 and K19 individually enhancing height by 53.7% and 51.2%, respectively, compared to the uninoculated drought control (Figure 7c).

## 4. Discussion

This study was aimed at isolating and assessing methylotrophic bacteria from maize rhizospheres in Kenya's semiarid regions to enhance plant growth and improve drought tolerance. Among the 100 isolates identified, varying levels of drought stress tolerance were observed at −1.3 MPa, indicating a diverse range of adaptive capabilities in these bacteria (Figure 1). This diversity is significant for selecting elite strains that can be applied sustainably to combat the effects of drought.

*Methylobacterium*, when compared to well-established genera such as *Bacillus* and *Rhizobium*, employs distinct mechanisms to support plant drought tolerance. Notably, its ability to utilize methanol from plant tissues allows it to thrive in the phyllosphere and rhizosphere under stress [40]. Five isolates (MK31, K2, K19, SH26, and SH33) demonstrated superior drought tolerance and were selected for further evaluation of their PGP properties under stress conditions and no-stress conditions as control. These isolates produced key growth-promoting substances such as IAA, GA, and EPS, which are potentially essential in plant stress responses. Isolate K2, in particular, showed high IAA production (Figure 2a), which is crucial for promoting root development, especially under drought conditions. This production of IAA by K2 suggests its potential role in enhancing root growth, which is critical for water uptake in stressed plants. The findings of this study corroborate previous research indicating that some species of *Methylobacterium*, such as *M. radiotolerans* ED5-9 and *Methylobacterium* sp. 2A, produce IAA, which has been shown to promote seed germination, boost plant development, and increase root length [14, 41].

It is noteworthy that while all isolates in this study produced PGP and drought tolerance bioactive compounds to varying extents, this variation can be attributed to strain-specific physiological traits, including differential regulation of phytohormone biosynthesis pathways under stress, variation in colonization efficiency, and distinct thresholds of drought tolerance. Some strains may sustain hormonal production only under mild stress, while others maintain activity under severe stress due to more robust metabolic systems or protective mechanisms like biofilm formation. These biological differences account for the inconsistency in responses observed across varying drought conditions. The increase in EPS production in response to higher drought stress levels highlights its role in improving soil structure and moisture retention, vital for plant survival in arid environments. EPS helps create a hydrated microenvironment around roots and microbial cells, shielding them from desiccation and enabling better nutrient exchange, thereby indirectly enhancing plant drought tolerance [42].

Isolate K19 emerged as a significant producer of GA (Figure 2c), even under severe drought stress, indicating its potential to maintain plant growth despite adverse conditions. The observed decline in GA production with increasing PEG 6000 concentration across all isolates is consistent with the understanding that drought stress can inhibit certain metabolic pathways. However, K19's sustained production of GA under stress suggests it has a resilient metabolic system, making it a strong candidate for use in biostimulant development. The antioxidant activities of the selected isolates were particularly noteworthy, with K2 and K19 exhibiting significant PO and PPO activities (Figure 3a,b). These enzymes play a critical role in mitigating oxidative stress in plants under drought conditions, helping to maintain cellular integrity and function. Additionally, the elevated CAT activity, especially in K19, further emphasizes its protective role in detoxifying reactive oxygen species generated during stress (Figure 3c). This capability is crucial for preventing cellular damage and ensuring plant survival under harsh conditions [43]. The elevated enzymatic activity observed in this study indicates that these *Methylobacterium* isolates may directly contribute to the plant's antioxidant defense system by either producing these enzymes themselves or triggering their upregulation in the host plant.

Osmoprotectants such as proline play a crucial role in enhancing plant tolerance to oxidative stress, a key mechanism for surviving drought conditions. All elite isolates demonstrated the ability to produce proline, though their efficiencies varied (Figure 3e). The accumulation of proline within plant tissues, facilitated by microbial inoculation, is known to safeguard essential plant proteins from denaturation and maintain their activity under stress conditions. In addition to stabilizing proteins and membranes, proline functions as a molecular chaperone and ROS scavenger, thus contributing to osmotic adjustment and stress signal transduction in drought-stressed plants. This protective effect significantly contributes to the plant's resilience against drought, as corroborated by previous research [44].

Additionally, SA, a vital phenolic compound, was produced by all tested isolates (Figure 3d). SA is indispensable for various plant processes, including photosynthesis, nitrogen fixation, and glycine betaine synthesis, all of which are critical for plant survival under adverse environmental conditions [45]. The consistent production of SA by these isolates underscores their potential as biostimulants to mitigate drought stress. This compound also acts as a signaling molecule that activates systemic acquired resistance (SAR) and modulates the expression of drought-responsive genes, thereby priming plants for enhanced stress tolerance. However, differences in SA levels among strains and stress conditions may reflect strain-dependent regulatory mechanisms, which in turn lead to varying degrees of induced drought resistance in the host plant.

The study also explored biofilm formation, revealing that *M. brachiatum* K2 maintained consistent biofilm levels under stress, whereas *M. radiotolerans* K19 increased biofilm production as stress intensified (Figure 5). Biofilm formation is a survival strategy that protects bacteria from environmental stressors and facilitates better microbial colonization of plant roots. The enhanced biofilm production by K19 under drought conditions suggests it may use this as a protective mechanism against dehydration, potentially improving plant–microbe interactions and contributing to plant resilience. In this way, biofilms not only protect the bacterial community but may also enhance root surface adhesion and facilitate the sustained release of growth-promoting compounds at the rhizosphere [46].

This study revealed the application of the two elite strains *M. brachiatum* K2 and *M. radiotolerans* K19 to maize seeds significantly enhanced both the emergence and development of seedlings. These elite strains promote plant development in many ways, such as directly improving seed germination (Table 2) and indirectly by reducing damaging seed microflora [47]. According to Wang et al. [48], the combination of different PGPB strains may increase drought tolerance even further. This research provides evidence that the coinoculation of the two elite strains enhances the maize biomass and height significantly under drought stress, making them promising candidates for use in sustainable agriculture, particularly in arid and semiarid regions.

## 5. Conclusion

This study successfully isolated and characterized drought-tolerant *Methylobacterium* spp. with multiple PGP traits. Among the isolates, strains K2 and K19 demonstrated superior performance under both normal and drought-stressed conditions. When applied individually at a concentration of 1 × 10^8^ CFU/mL, both K2 and K19 significantly enhanced maize germination rate, seedling vigor, plant height, and shoot biomass compared to untreated controls. Notably, the combined application of K2 and K19 resulted in the most pronounced improvements across all measured parameters, indicating a synergistic effect in promoting growth and mitigating drought stress.

Based on these findings, it is recommended to use K2 and K19 strains in combination as an effective biostimulant formulation to enhance drought resilience and overall productivity in maize cultivation. Further field trials are still needed to validate their efficacy under diverse environmental conditions and support their integration into sustainable agricultural practices, especially in drought-prone regions.

## Figures and Tables

**Table 1:** Isolates and associated BLAST strains using partial 16S ribosomal gene sequencing.

**Isolates code**	**Accession number**	**NCBI best match**	**Sequence length**	**E** ** -value**	**% query coverage**	**% similarity with closet match**
K2	PP708908	*Methylobacterium brachiatum* K2	1447	0.0	100	99.03
K19	PP708910	*Methylobacterium radiotolerans* K19	1436	0.0	100	99.25

**Table 2:** Effect of microbial seed inoculation on vigor index, seedling length, and germination rate.

**Seed treatments**			**Vigor index**	**Total seedling length (cm)**	**Germination (%)**
	**Strains**	**Stress**			
T1	−	−	877.30 ± 5.3c	9.40 ± 0.05de	93.33 ± 3.85ab
T2	−	+	65.31 ± 1.79a	4.20 ± 0.12g	15.55 ± 2.22e
T3	K2	−	1180.00 ± 5.77b	11.80 ± 0.06c	100.00 ± 0.00a
T4	K2	+	442.63 ± 9.3f	8.30 ± 0.17f	53.33 ± 3.85d
T5	K19	−	1231.95 ± 16.16b	13.20 ± 0.17b	93.33 ± 3.85ab
T6	K19	+	652.63 ± 4.23e	8.90 ± 0.06ef	73.33 ± 6.65c
T7	K2 + K19	−	1480.00 ± 11.54a	14.80 ± 0.12a	100.00 ± 0.00a
T8	K2 + K19	+	768.00 ± 18.47d	9.60 ± 0.23d	80.00 ± 3.95bc

*Note:* Different lowercase letters denote that the Tukey test was done at a significance level of 0.05.

## Data Availability

The article contains data supporting this study; further inquiries can be directed to the corresponding author.
