# Supplementary material for: Methylobacterium spp. Isolated From Semiarid Soils Promote Growth and Drought Tolerance in Maize in Kenya
Source: Int J Microbiol. 2025 Sep 8;2025:7442350. doi: 10.1155/ijm/7442350 (PMC12436008; doi:10.1155/ijm/7442350)
Supplement: Supporting Information — Additional supporting information can be found online in the Supporting Information section. Table S1: The OD600 values of the bacterial isolates at −1.3 MPa osmotic pressure. [file 7442350.f1.docx]

Table S1. The OD 600 values of the bacterial isolates at ( -1.3MPa) osmotic pressure.

| No | Isolate | OD600 Value | Mean Error | Classification |
| --- | --- | --- | --- | --- |
| 1 | MK31 | 0.55 | 0.02 | Highly Tolerant |
| 2 | K2 | 0.67 | 0.03 | Highly Tolerant |
| 3 | K19 | 0.63 | 0.02 | Highly Tolerant |
| 4 | SH26 | 0.58 | 0.02 | Highly Tolerant |
| 5 | SH33 | 0.56 | 0.01 | Highly Tolerant |
| 6 | SH5 | 0.48 | 0.02 | Tolerant |
| 7 | SH7 | 0.45 | 0.02 | Tolerant |
| 8 | SH11 | 0.47 | 0.03 | Tolerant |
| 9 | SH12 | 0.49 | 0.02 | Tolerant |
| 10 | SH18 | 0.46 | 0.03 | Tolerant |
| 11 | SH19 | 0.44 | 0.02 | Tolerant |
| 12 | SH21 | 0.43 | 0.03 | Tolerant |
| 13 | SH9 | 0.42 | 0.02 | Tolerant |
| 14 | MK26 | 0.48 | 0.01 | Tolerant |
| 15 | MK4 | 0.47 | 0.02 | Tolerant |
| 16 | MK6 | 0.49 | 0.03 | Tolerant |
| 17 | MK8 | 0.44 | 0.02 | Tolerant |
| 18 | MK12 | 0.46 | 0.03 | Tolerant |
| 19 | MK14 | 0.45 | 0.02 | Tolerant |
| 20 | MK19 | 0.42 | 0.03 | Tolerant |
| 21 | K3 | 0.46 | 0.02 | Tolerant |
| 22 | K5 | 0.48 | 0.01 | Tolerant |
| 23 | K8 | 0.44 | 0.02 | Tolerant |
| 24 | K10 | 0.43 | 0.02 | Tolerant |
| 25 | K13 | 0.42 | 0.03 | Tolerant |
| 26 | K24 | 0.49 | 0.02 | Tolerant |
| 27 | K7 | 0.41 | 0.03 | Tolerant |
| 28 | SH1 | 0.35 | 0.02 | Sensitive |
| 29 | SH4 | 0.38 | 0.01 | Sensitive |
| 30 | SH8 | 0.36 | 0.02 | Sensitive |
| 31 | SH20 | 0.37 | 0.03 | Sensitive |
| 32 | SH27 | 0.39 | 0.01 | Sensitive |
| 33 | SH28 | 0.31 | 0.02 | Sensitive |
| 34 | SH29 | 0.35 | 0.02 | Sensitive |
| 35 | SH30 | 0.32 | 0.03 | Sensitive |
| 36 | SH35 | 0.38 | 0.01 | Sensitive |
| 37 | MK1 | 0.36 | 0.02 | Sensitive |
| 38 | MK7 | 0.33 | 0.02 | Sensitive |
| 39 | MK11 | 0.37 | 0.01 | Sensitive |
| 40 | MK13 | 0.34 | 0.02 | Sensitive |
| 41 | MK16 | 0.32 | 0.03 | Sensitive |
| 42 | MK17 | 0.38 | 0.02 | Sensitive |
| 43 | MK23 | 0.39 | 0.01 | Sensitive |
| 44 | MK27 | 0.31 | 0.02 | Sensitive |
| 45 | K9 | 0.36 | 0.02 | Sensitive |
| 46 | K14 | 0.35 | 0.02 | Sensitive |
| 47 | K15 | 0.38 | 0.01 | Sensitive |
| 48 | K16 | 0.33 | 0.02 | Sensitive |
| 49 | K20 | 0.34 | 0.02 | Sensitive |
| 50 | K23 | 0.37 | 0.02 | Sensitive |
| 51 | K25 | 0.39 | 0.01 | Sensitive |
| 52 | SH2 | 0.35 | 0.02 | Sensitive |
| 53 | SH6 | 0.39 | 0.01 | Sensitive |
| 54 | SH15 | 0.36 | 0.02 | Sensitive |
| 55 | SH16 | 0.38 | 0.02 | Sensitive |
| 56 | SH22 | 0.34 | 0.03 | Sensitive |
| 57 | SH23 | 0.22 | 0.02 | Highly Sensitive |
| 58 | SH24 | 0.21 | 0.03 | Highly Sensitive |
| 59 | SH25 | 0.29 | 0.01 | Highly Sensitive |
| 60 | SH34 | 0.28 | 0.02 | Highly Sensitive |
| 61 | SH37 | 0.23 | 0.02 | Highly Sensitive |
| 62 | SH10 | 0.27 | 0.02 | Highly Sensitive |
| 63 | SH17 | 0.22 | 0.02 | Highly Sensitive |
| 64 | MK3 | 0.25 | 0.03 | Highly Sensitive |
| 65 | MK9 | 0.27 | 0.02 | Highly Sensitive |
| 66 | MK18 | 0.24 | 0.02 | Highly Sensitive |
| 67 | MK21 | 0.26 | 0.02 | Highly Sensitive |
| 68 | MK22 | 0.29 | 0.01 | Highly Sensitive |
| 69 | MK25 | 0.28 | 0.02 | Highly Sensitive |
| 70 | MK29 | 0.22 | 0.03 | Highly Sensitive |
| 71 | MK30 | 0.24 | 0.03 | Highly Sensitive |
| 72 | MK32 | 0.21 | 0.02 | Highly Sensitive |
| 73 | MK33 | 0.23 | 0.02 | Highly Sensitive |
| 74 | K1 | 0.25 | 0.02 | Highly Sensitive |
| 75 | K4 | 0.24 | 0.03 | Highly Sensitive |
| 76 | K11 | 0.29 | 0.02 | Highly Sensitive |
| 77 | K17 | 0.28 | 0.02 | Highly Sensitive |
| 78 | K18 | 0.26 | 0.02 | Highly Sensitive |
| 79 | K28 | 0.27 | 0.03 | Highly Sensitive |
| 80 | K26 | 0.22 | 0.02 | Highly Sensitive |
| 81 | SH3 | 0.27 | 0.02 | Highly Sensitive |
| 82 | SH14 | 0.28 | 0.02 | Highly Sensitive |
| 83 | SH13 | 0.29 | 0.01 | Highly Sensitive |
| 84 | SH31 | 0.26 | 0.02 | Highly Sensitive |
| 85 | MK5 | 0.24 | 0.03 | Highly Sensitive |
| 86 | MK10 | 0.23 | 0.02 | Highly Sensitive |
| 87 | MK15 | 0.27 | 0.02 | Highly Sensitive |
| 88 | MK20 | 0.28 | 0.02 | Highly Sensitive |
| 89 | MK2 | 0.25 | 0.02 | Highly Sensitive |
| 90 | K12 | 0.22 | 0.03 | Highly Sensitive |
| 91 | K22 | 0.24 | 0.02 | Highly Sensitive |
| 92 | K6 | 0.21 | 0.03 | Highly Sensitive |
| 93 | K27 | 0.23 | 0.02 | Highly Sensitive |
| 94 | K29 | 0.22 | 0.03 | Highly Sensitive |
| 95 | SH32 | 0.21 | 0.02 | Highly Sensitive |
| 96 | SH36 | 0.23 | 0.02 | Highly Sensitive |
| 97 | MK24 | 0.24 | 0.03 | Highly Sensitive |
| 98 | MK28 | 0.25 | 0.02 | Highly Sensitive |
| 99 | K21 | 0.29 | 0.02 | Highly Sensitive |
| 100 | K30 | 0.23 | 0.03 | Highly Sensitive |

The presented data represent average measurements obtained from triplicate experiments, each with its standard error (± S.E.). The isolates were classified according to their optical density (OD600) values, which were used to assess their degree of drought sensitivity. Isolates with an OD600 value less than 0.3 were categorized as highly sensitive, those with an OD600 value between 0.3 and 0.39 were classified as sensitive, isolates with an OD600 value between 0.4 and 0.5 were considered tolerant, and isolates with an OD600 value greater than 0.5 were classified as completely tolerant.
